# Blood-Derived Plasma Protein Biomarkers for Alzheimer’s Disease in Han Chinese

**DOI:** 10.3389/fnagi.2018.00414

**Published:** 2018-12-17

**Authors:** Zaohuo Cheng, Jiajun Yin, Hongwei Yuan, Chunhui Jin, Fuquan Zhang, Zhiqiang Wang, Xiaowei Liu, Yue Wu, Tao Wang, Shifu Xiao

**Affiliations:** ^1^Wuxi Mental Health Center, Nanjing Medical University, Wuxi, China; ^2^Shanghai Mental Health Center, Shanghai Jiao Tong University School of Medicine, Shanghai, China

**Keywords:** Alzheimer’s disease, biomarker, plasma protein, early diagnosis, Han Chinese

## Abstract

It is well known that Alzheimer’s disease (AD) is one of the most common progressive neurodegenerative diseases; it begins gradually, and therefore no effective medicine is administered in the beginning. Thus, early diagnosis and prevention of AD are crucial. The present study focused on comparing the plasma protein changes between patients with AD and their healthy counterparts, aiming to explore a specific protein panel as a potential biomarker for AD patients in Han Chinese. Hence, we recruited and collected plasma samples from 98 AD patients and 101 elderly healthy controls from Wuxi and Shanghai Mental Health Centers. Using a Luminex assay, we investigated the expression levels of fifty plasma proteins in these samples. Thirty-two out of 50 proteins were found to be significantly different between AD patients and healthy controls (*P* < 0.05). Furthermore, an eight-protein panel that included brain-derived neurotrophic factor (BDNF), angiotensinogen (AGT), insulin-like growth factor binding protein 2 (IGFBP-2), osteopontin (OPN), cathepsin D, serum amyloid P component (SAP), complement C4, and prealbumin (transthyretin, TTR) showed the highest determinative score for AD and healthy controls (all *P* = 0.00). In conclusion, these findings suggest that a combination of eight plasma proteins can serve as a promising diagnostic biomarker for AD with high sensitivity and specificity in Han Chinese populations; the eight plasma proteins were proven important for AD diagnosis by further cross-validation studies within the AD cohort.

## Introduction

Recently, the Centers for Disease Control and Prevention in the United States reported a 54.5% increase from the 1999 rate of 16.5 deaths per 100,000 patients with Alzheimer’s disease (AD) (Taylor et al., 2017), which is one of the most common progressive neurodegenerative diseases and is characterized by the interaction of both genetic and environmental factors, resulting in memory dysfunction and behavioral changes (Hooli and Tanzi, 2009). In 2013, the number of older people in China was almost 200 million, and the proportion of the population that was aged 65 years and older increased to 14.3% (Sun et al., 2015). The World Alzheimer Report 2015 updated the estimates of the global prevalence, China is the region with the most people living with dementia (9.5 million), and the prevalence for the population aged 60 and older is 6.19% (Prince et al., 2015; Wu et al., 2018). Currently, disease-modifying therapy and an ideal diagnostic tool for AD are largely lacking, severely influencing patients’ quality of life and leading to a heavy financial burden for patients’ families.

Preclinical Alzheimer’s, the newly defined disease stage, demonstrates that brain changes are progressively initiated 10 to 20 years before the onset of dementia symptoms (Bateman et al., 2012). Thus, identifying biomarkers for preclinical Alzheimer’s contributed to the early recognition and prediction of the progression of AD. Notably, the diagnostic tool should be inexpensive, easy to perform, and non-invasive (The Ronald and Nancy Reagan Research Institute of the Alzheimer’s Association and the National Institute on Aging Working Group, 1998). Recent scientific evidence suggests some potential diagnostic biomarkers such as β-amyloid (Aβ) (Blennow et al., 2015) and tau (Meredith et al., 2013) accumulation in cerebrospinal fluid (CSF), Pittsburgh compound B positron emission tomography (PiB-PET) (Leuzy et al., 2015), and peripheral blood protein (Zhao et al., 2015) and microRNA (miRNA) expression (Galimberti et al., 2014), among which blood-derived biomarkers have been extensively studied due to their less invasive source (Blennow, 2017; Kitamura et al., 2017; O’Bryant et al., 2017). Although previous studies have demonstrated plasma protein profiles may be a valuable diagnostic biomarker for the early stage of AD, the findings have not been widely replicated in different races (Kiddle et al., 2014; Shi et al., 2018).

Therefore, based on these findings, the present study focuses on plasma protein differences in healthy individuals and patients with AD, exploring a specific protein panel as a potential diagnostic biomarker for AD patients in Han Chinese.

## Materials and Methods

### Study Population

We recruited 1,105 older people aged 56–95 years from 2015 to 2017. Eventually, 98 AD patients diagnosed with the criteria of the National Institute of Neurological and Communicative Disorders and Stroke and the Alzheimer’s disease and Related Disorders Association (NINCDS-ADRDA) were recruited from Wuxi and Shanghai Mental Health Centers. The cognitive function of these participants was assessed by two skilled professional psychiatrists using the Mini-Mental State Examination (MMSE). The absence of depression was documented on the basis of a score of 10 or less on the Hamilton Rating Scale for Depression (HAMD). A brain computed tomography (CT) or magnetic resonance imaging (MRI) scan excluded other structural brain diseases, and neurologic examination showed no significant abnormalities. A total of 101 elderly healthy subjects were recruited through advertisements, and their demographic characteristics were carefully recorded.

### Exclusion Criteria

Patients with any dementia other than AD, such as vascular dementia, dementia with Lewy bodies, frontotemporal dementia or Parkinson’s disease, were excluded. In addition, a history of stroke or cerebrovascular disease, bone marrow transplantation, major psychiatric disorder, and a history of alcohol or drug abuse were causes for exclusion from the study.

### Study Design

Plasma samples from AD and elderly healthy controls from Shanghai and Wuxi in China were obtained. In total, we examined plasma samples from 199 subjects: 98 with AD and 101 elderly controls with no dementia. Multiple protein differences were explored using Luminex xMAP technology. Statistical analysis was performed to assess the relative importance of these protein biomarkers for the diagnosis of AD.

This study was approved by the Ethics Committees of the Wuxi Health Mental Center. Either patients or their guardians signed informed consent. If participants failed to fill out the consent form more than twice, their guardians were asked to fill out the consent form on the patients’ behalf.

### Luminex Assays

Luminex xMAP technology (Austin, TX, United States) uses a solid phase approach to analyze multiple proteins. In brief, the xMAP technology is a flow cytometric-based platform that uses microspheres inserted with a ratio of two different fluorescent dyes. In theory, up to 100 differently colored beads can be generated with a theoretical multiplex capacity of up to 100 assays per well of a 96-well plate. The capture antibody is covalently coupled to the bead, and immunoassays are run under standard sandwich immunoassay formats (Hye et al., 2014).

The Luminex kits were obtained from Millipore (Billerica, MA, United States) and the assays were performed according to the manufacturer’s instructions (Soares et al., 2009). Eleven Milliplex MAP multiplex panels covering 50 proteins (96-well plate format; EMD Millipore) were utilized: cat.# HCYTOMAG-60K (7-plex); cat.# HIGFBMAG-53K (2-plex); cat.# HMHEMAG-34K (2-plex); cat.# HMMP2MAG-55K (2-plex); cat.# HNDG1MAG-36K (7-plex); cat.# HNDG2MAG-36K (6-plex); cat.# HNDG3MAG-36K (10-plex); cat.# HND2MAG-39K (3-plex); cat.# HND3MAG-39K (7-plex); cat.# SKINMAG-50K (1-plex); and cat.# HKI6MAG-99K (3-plex). Properly diluted plasma samples were incubated with the antibody-coupled microspheres and then with biotinylated detection antibody before the addition of streptavidin-phycoerythrin. The captured bead complexes were measured with a FLEXMAP 3D system (Luminex Corporation, Austin, TX, United States) using the following instrument settings: events/bead, 50; sample size, 50 μL; discriminator gate, 8000–15,000. The raw data (mean fluorescence intensity) were collected and further processed for calculating protein concentration (Zhi et al., 2014; Al-Daghri et al., 2018).

### Data Processing

Quality checks (QC) based on standard curve linearity, intraassay coefficient of variation, interassay coefficient of variation for reference sample, and percentage of missing data were performed to examine the performance of each assay followed by measuring median fluorescent intensity (MFI) using xPONENT 5.1 (Luminex Corporation). This was further exported into Milliplex Analyst 5.1 (VigeneTech, United States) to calculate protein concentrations by a five-parameter logistic fit. Afterward, all analytes were subjected to the statistical analysis.

### Statistical Analysis

All analysis results were expressed as the mean ± SD. Statistical analyses were performed using the Statistical Package for the Social Sciences software version 20.0 (SPSS Inc., Chicago, IL, United States). Pearson’s chi-square test was used to compare gender between control subjects and AD patients. All 50 blood protein levels were non-normally distributed, and subsequently underwent ln or square root transformation. The results of transforming the variables are displayed in the table. An independent-sample *t*-test was used to compare age, education and MMSE score and overall protein differences between control subjects and AD patients. Discriminant analysis was performed to assess the relative importance of these biomarkers in classifying AD and controls. In the case of the stepwise method, Wilk’s lambda method was used to build the prediction model. The discriminant analysis used a partial *F*-test (F to enter 3.84; F to remove 2.71) and a stepwise method (maximum number of steps = 64) to sequentially incorporate the set of 32 significant variables into the canonical discriminant function. To check the reliability of our analysis, leave-one-out cross-validation was used. Receiver operating characteristic (ROC) analyses were conducted under the non-parametric distribution assumption for standard error of area to determine the performance of the models for discriminating AD from controls.

## Results

### Study Participants

The demographic and clinical characteristics of healthy controls and AD patients are presented in Table 1. Briefly, the mean age of AD patients and their healthy counterparts were 78.76 ± 8.06 and 78.33 ± 7.30 years, respectively. No significant difference was found in age, gender or education between these two groups. As expected, patients with AD had significantly lower MMSE scores than healthy controls (*P* = 0.000).

**Table 1:** Characteristics of AD patients and control subjects.

	Controls (*n* = 101)	AD (*n* = 98)	χ^2^/*t*-test	*P*-value
Gender (M/F)	35/66	34/64	0.000	0.995
Age (years)	78.33 ± 7.30	78.76 ± 8.06	-0.393	0.695
Education (years)	5.77 ± 4.77	5.00 ± 4.37	1.175	0.241
MMSE score	25.70 ± 2.90	8.62 ± 7.58	20.783	0.000^*^


*Chi-square was used for the gender comparison. Independent-sample t-test was used for age, education, and MMSE score. ^∗^P < 0.05 indicated statistical significance.*

### Differentially Expressed Protein

In this study, we applied Luminex assay technology to determine the expression profiles of 50 proteins in the plasma from AD patients and healthy controls. Of the fifty candidates, thirty-two proteins were found to be differentially expressed, with statistical significance between AD patients and healthy controls (*P* < 0.05) (Table 2).

**Table 2:** Blood protein levels of AD patients and control subjects.

	Data conversion	AD (*n* = 98)	Controls (*n* = 101)	*t*-Test	*P*-value
G-CSF (pg/ml)	Ln (x+1)	3.510 ± 1.166	3.052 ± 1.097	2.584	0.005^**^
IL-10 (pg/ml)	Ln (x+1)	1.039 ± 0.800	0.759 ± 0.730	2.578	0.011^*^
MCP (pg/ml)	Ln (x+1)	1.106 ± 1.715	1.100 ± 1.768	0.026	0.980
IL-1a (pg/ml)	Ln (x+1)	2.044 ± 1.567	1.659 ± 1.578	1.726	0.086
IL-3 (pg/ml)	Ln (x+1)	0.239 ± 0.276	0.147 ± 0.143	2.953	0.004^**^
IL-8 (pg/ml)	Ln (x+1)	1.896 ± 1.260	1.726 ± 1.205	0.972	0.332
MIP (pg/ml)	Ln (x+1)	0.511 ± 0.811	0.528 ± 0.825	-0.152	0.880
IGFBP-2 (ng/ml)	Ln (x+1)	4.933 ± 1.085	4.122 ± 1.090	5.263	0.000^***^
IGFBP-6 (ng/ml)	Ln (x)	6.179 ± 0.461	6.169 ± 0.502	0.137	0.891
B-2 Microglobulin (ng/ml)	Ln (x)	8.064 ± 0.874	7.529 ± 0.770	4.580	0.000^***^
Clusterin (ng/ml)	SQRT (x)	878.97 ± 162.09	729.87 ± 183.25	6.069	0.000^***^
Cystatin C (ng/ml)	SQRT (x)	30.248 ± 7.670	23.610 ± 8.306	5.852	0.000^***^
PP (pg/ml)	Ln (x)	4.802 ± 0.971	4.667 ± 0.791	1.084	0.280
TNFa (pg/ml)	SQRT (x)	2.778 ± 0.731	2.622 ± 0.724	1.511	0.132
MMP-2 (pg/ml)	Ln (x)	12.046 ± 0.286	11.976 ± 0.232	1.882	0.061
MMP-9 (pg/ml)	Ln (x)	10.37 ± 0.700	10.55 ± 0.589	-1.927	0.055
CP (ng/ml)	SQRT (x)	551.21 ± 229.25	557.25 ± 196.47	-0.200	0.842
AGP (ng/ml)	Ln (x)	13.155 ± 1.944	13.475 ± 1.200	-1.394	0.163
HP (ng/ml)	SQRT (x)	1434.3 ± 926.7	1714.9 ± 1025.6	-2.023	0.044^*^
sSOD1 (ng/ml)	Ln (x)	5.440 ± 0.656	5.443 ± 0.498	-0.031	0.975
AGT (ng/ml)	Ln (x)	4.476 ± 1.993	6.470 ± 1.729	-7.544	0.000^***^
Fetuin A (ng/ml)	SQRT (x)	371.97 ± 45.32	377.39 ± 55.23	-0.755	0.451
sSOD2 (ng/ml)	Ln (x)	3.773 ± 0.476	3.640 ± 0.352	2.232	0.027^*^
Contactin-1 (ng/ml)	SQRT (x)	11.44 ± 1.982	11.29 ± 1.570	0.592	0.554
Kallikrein-6 (ng/ml)	SQRT (x)	2.806 ± 0.628	2.555 ± 0.452	3.252	0.001^**^
OPN (ng/ml)	SQRT (x)	7.060 ± 1.489	5.874 ± 1.248	6.098	0.000^***^
a2-Macroglobulin (ng/ml)	SQRT (x)	1299.5 ± 304.59	1146.9 ± 329.78	3.388	0.001^**^
APO A1 (ng/ml)	SQRT (x)	625.61 ± 175.59	609.57 ± 250.79	0.521	0.603
APO CIII (ng/ml)	SQRT (x)	411.28 ± 113.22	368.82 ± 137.12	2.385	0.018^*^
APO E (ng/ml)	SQRT (x)	289.40 ± 71.73	253.24 ± 90.32	3.133	0.002^**^
Complement C3 (ng/ml)	SQRT (x)	184.42 ± 76.35	208.99 ± 73.32	-2.331	0.021^*^
Prealbumin (ng/ml)	SQRT (x)	457.61 ± 113.94	411.31 ± 128.43	2.687	0.008^**^
Complement factor H (ng/ml)	SQRT (x)	583.95 ± 115.00	502.41 ± 165.47	4.047	0.000^***^
CRP (ng/ml)	Ln (x)	9.255 ± 2.643	9.580 ± 1.352	-1.086	0.279
A1-Antitrypsin (ng/ml)	Ln (x)	7.649 ± 1.340	8.151 ± 1.190	-2.792	0.006^**^
PEDF (ng/ml)	SQRT (x)	61.96 ± 23.23	63.61 ± 17.57	-0.565	0.572
SAP (ng/ml)	Ln (x)	10.812 ± 2.551	11.656 ± 1.368	-2.921	0.004^**^
MIP-4 (ng/ml)	Ln (x)	4.140 ± 0.784	4.011 ± 0.637	1.277	0.203
Complement C4 (ng/ml)	SQRT (x)	156.81 ± 72.86	116.06 ± 49.76	4.594	0.000^***^
BDNF (pg/ml)	Ln (x)	8.006 ± 1.219	6.818 ± 0.942	7.705	0.000^***^
Cathepsin D (pg/ml)	SQRT (x)	608.92 ± 178.92	479.43 ± 154.10	5.476	0.000^***^
sICAM-1 (pg/ml)	SQRT (x)	365.19 ± 69.65	300.29 ± 84.30	5.910	0.000^***^
MPO (pg/ml)	SQRT (x)	12.080 ± 1.491	11.519 ± 1.494	2.649	0.009^**^
PDGF-AA (pg/ml)	Ln (x)	7.212 ± 0.969	6.322 ± 0.756	7.206	0.000^***^
RANTES (pg/ml)	Ln (x)	10.122 ± 1.025	9.138 ± 1.030	6.751	0.000^***^
NCAM (pg/ml)	SQRT (x)	608.35 ± 96.051	505.86 ± 126.59	6.446	0.000^***^
PDGF-AB/BB (pg/ml)	Ln (x)	8.270 ± 1.161	7.789 ± 0.621	7.185	0.000^***^
sVCAM-1 (pg/ml)	SQRT (x)	956.61 ± 185.54	778.87 ± 215.38	6.215	0.000^***^
PAI-1 Total (pg/ml)	SQRT (x)	366.67 ± 117.04	331.95 ± 124.71	2.024	0.044^*^
HSA (μg/ml)	SQRT (x)	208.60 ± 40.69	192.74 ± 51.37	2.410	0.017^*^


*Significant P-value (^∗^P < 0.05, ^∗∗^P < 0.01, ^∗∗∗^P < 0.001).*

### Stepwise Discriminant Function Analysis

Afterward, we performed a stepwise discriminant function analysis to further determine how effectively AD patients and healthy controls can be distinguished based on the expressed protein levels and to assess the differential contribution to the diagnosis. Of the 32 significant plasma markers, a feature group of eight most discriminative proteins, including brain-derived neurotrophic factor (BDNF), angiotensinogen (AGT), insulin-like growth factor binding protein 2 (IGFBP-2), osteopontin (OPN), cathepsin D, serum amyloid P component (SAP), complement C4, and prealbumin (transthyretin, TTR), was sorted out by stepwise discriminant analysis (Table 3, all *P* = 0.00), indicating their potential contributions to diagnosis. To detect whether this 8-protein panel was efficient in differentiating AD from healthy controls, we carried out both original- and cross-validation, correctly classifying 86.7 and 84.7% of the cases, respectively (Table 4).

**Table 3:** Summary of canonical discriminant analysis (stepwise method).

Step	Entered variable	Wilk’s lambda	Exact F	*P*-value	Standardized coefficients
1	BDNF	0.768	59.363	0.000	0.355
2	AGT	0.651	52.431	0.000	-0.459
3	IGFBP-2	0.557	51.731	0.000	0.395
4	OPN	0.522	44.492	0.000	0.330
5	Cathepsin D	0.490	40.134	0.000	0.286
6	SAP	0.453	38.665	0.000	-0.748
7	Complement C4	0.410	39.311	0.000	0.549
8	Prealbumin	0.398	35.967	0.000	0.258


*At each step, the variable that minimizes the overall Wilk’s lambda is entered: a. Maximum number of steps is 64; b. Minimum partial F to enter is 3.84; c. Maximum partial F to remove is 2.71; d. F level, tolerance, or VIN insufficient for further computation.*

**Table 4:** Discriminant classification results^b,c^.

Clinical diagnosis		Predicted group membership		Total
		Controls	AD	
Original	Controls	89 (88.1%)	12 (11.9%)	101
	AD	13 (13.3%)	85 (86.7%)	98
	Total	107	92	199
Cross-validated^a^	Controls	87 (86.1%)	14 (13.9%)	101
	AD	15 (15.3%)	83 (84.7%)	98
	Total	106	93	199


*^a^Cross validation was performed only for those cases in the analysis. In cross validation, each case is classified by the functions derived from all cases other than that case. ^b^86.7% of original grouped cases correctly classified. ^c^84.7% of cross-validated grouped cases correctly classified.*

### The Classification Performance of the 8-Protein Panel

**FIGURE 1:**
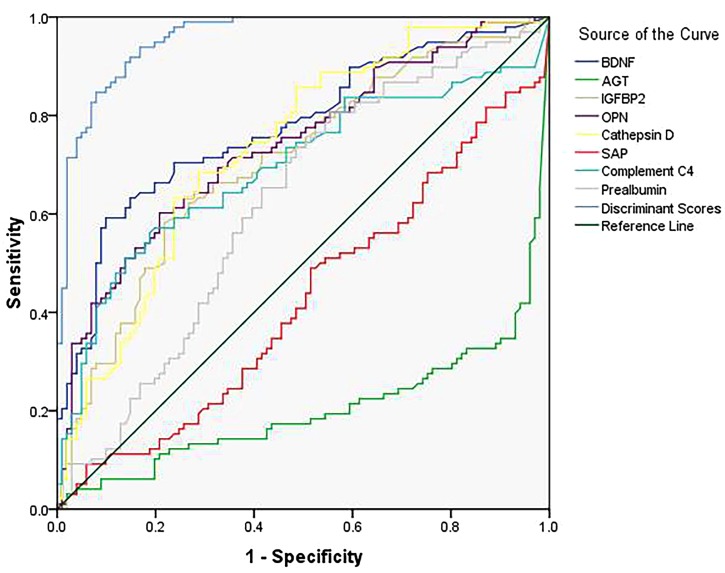
Receiver-operating characteristic (ROC) curve for the 8-protein panel and each protein.

Furthermore, we decided the classification performance of the eight-protein panel and each biomarker by calculating the discriminant score (Table 5) and receiver operating characteristic (ROC) curve (Figure 1), resulting in an 87.4% correct classification for AD and control subjects with high sensitivity (86.7%) and specificity (88.1%), which suggested that the combination of these eight differentially expressed plasma proteins produced the most accurate results in a threshold classification.

**Table 5:** Summary of ROC curve analysis.

	AUC				Sensitivity	Specificity	Correct classification
	Area	Standard error^a^	Asymptotic significance^b^	95% CI			
BDNF	0.776	0.033	0.000	0.711∼0.841	0.704	0.743	72.4%
AGT	0.203	0.034	0.000	0.138∼0.269	0.776	0.634	70.4%
IGFBP-2	0.707	0.037	0.000	0.635∼0.779	0.673	0.634	65.3%
OPN	0.739	0.035	0.000	0.671∼0.808	0.643	0.723	68.3%
Cathepsin D	0.735	0.035	0.000	0.666∼0.804	0.571	0.762	66.8%
SAP	0.425	0.041	0.067	0.345∼0.504	0.449	0.634	54.3%
Complement C4	0.694	0.038	0.000	0.618∼0.769	0.643	0.644	64.3%
Prealbumin	0.619	0.038	0.004	0.541∼0.697	0.653	0.574	61.3%
Discriminant Score	0.958	0.012	0.000	0.934∼0.982	0.867	0.881	87.4%


*^a^Under the non-parametric assumption. ^b^Null hypothesis: true area = 0.5.*

## Discussion

To date, disease-modifying treatments have not been successfully developed for AD. Continuous clinical trials for novel drugs have failed, suggesting early diagnosis and prevention of AD is crucial for postponing the progression of disease effectively. Approximately 500 mL of cerebrospinal fluid (CSF), which is in direct contact with the extracellular space of the brain, is absorbed into the blood daily (Davidsson et al., 2002; Davidsson and Sjogren, 2006; Hye et al., 2006). Plasma contains multiple biological components, including proteins, peptides, lipids, and metabolites, which also effectively reflect physiological activity and pathology in the central nervous system (CNS). To our knowledge, this is the first study to investigate potential plasma protein biomarkers in the Han Chinese population using a high-throughput multiplexed xMAP Luminex assay. Our present findings suggest that a set of eight plasma proteins (BDNF, AGT, IGFBP-2, OPN, cathepsin D, SAP, complement C4, and TTR) serves as a putative predictor panel for AD diagnosis with high sensitivity and specificity. Importantly, these proteins have been considered to be interesting and potentially significant in AD disease pathology in previous studies (Phillips et al., 1991; Iadecola and Davisson, 2008; Carecchio and Comi, 2011; Daborg et al., 2012; Mold et al., 2012; Tian et al., 2014; Willette et al., 2015; Buxbaum and Johansson, 2017).

Recently, blood-derived biomarkers for AD have been widely considered for being relatively painless, inexpensive and having diagnostic accuracy. Using predictive analysis of microarrays, a previous study demonstrated that a panel of 18 plasma proteins (CCL18, CCL15, CCL7, CXCL8, ICAM-1, TRAIL-R4, G-CSF, GDNF, EGF, CCL5, M-CSF, IL-3, IL-1α, TNF-α, PDGF-BB, IL-11, ANG-2, and IGFBP-6) achieved a diagnostic accuracy of 90% in distinguishing AD and mild cognitive impairment (MCI) (Ray et al., 2007), which was reported to be unable to distinguish patients with AD from the populations through enough diagnostic precision (Bjorkqvist et al., 2012). In addition, it also failed to reach comparable accuracy in discriminating patients with AD and healthy controls using 16 and 8 plasma proteins derived from this 18-protein panel, respectively (Soares et al., 2009; Marksteiner et al., 2011), which might be attributed to only three of these 18 plasma proteins being differentially expressed between AD and healthy controls in an independent replication study. Afterward, Thambisetty et al. (2008) also performed a subsequent study to investigate 26 proteins that had been identified as potential AD biomarkers, including the 18-protein panel reported by Ray et al. (2007). Although only two proteins were found to be significantly different between AD and controls, they identified that a 10-protein panel (TTR, CLU, cystatin C, A1AcidG, ICAM-1, CC4, pigment epithelium-derived factor, A1AT, RANTES, and ApoC3) could predict the progression of MCI to AD with high diagnostic accuracy. However, of these proteins, ICAM-1 was a unique protein that overlapped with Ray’s study. O’Bryant and colleagues used a panel from RBM to identify a list of 30 biomarkers to detect AD (O’Bryant et al., 2010, 2011).

It is well known that the pathological mechanism of AD is diverse. The fifty proteins selected in the present study, including those proteins that were reported in previous studies, are involved in the various signaling pathways associated with AD, including the immune response (Heneka et al., 2015), inflammatory (Harrison, 2013; Heneka et al., 2015) and antioxidant (Andrieu et al., 2015) processes, and metabolism (Arnoldussen et al., 2014; Counts et al., 2017). Of this 8-protein panel, three proteins, CC4, TTR, and IGFBP-2, overlapped with previous studies (Bennett et al., 2012; Uchida et al., 2015; McLimans et al., 2017) and affect immunology, Aβ fibril formation, DNA synthesis, and cell proliferation and death in AD.

The complement system is considered to be highly involved in the inflammatory response as a powerful component of innate immunity, consisting of more than 30 fluid-phase and cell-associated proteins as well as a wide range of specific receptors that interact to trigger the inflammatory response (Ricklin et al., 2010; Wagner and Frank, 2010). Recent genome-wide association studies (GWAS) have identified the association of complement receptor 1 with AD (Harold et al., 2009; Lambert et al., 2009; Seshadri et al., 2010; Naj et al., 2011). However, whether these complement molecules have any diagnostic value has not been fully elucidated. Elevated levels of complement 3 and 4 (C3 and C4) in cerebrospinal fluid (CSF) were found in AD, compared with MCI, patients (Daborg et al., 2012). Moreover, increased plasma levels of C4a protein were found in the plasma of AD patients (Bennett et al., 2012). Consistently, complement C4 was highly expressed in the plasma of AD participants in this study. We further showed that complement C4 had 64.3% accuracy for distinguishing AD from healthy individuals with 64.3% sensitivity and 64.4% specificity, indicating that complement C4 alone was inadequate as a biomarker for AD diagnosis.

Transthyretin (TTR) is a transport protein, also known as prealbumin, which has been found in cerebrospinal fluid (CSF) as an Aβ-binding protein and suppresses the toxicity of oligomers. Thus, the sequester protein, as an efficient inhibitor of Aβ fibril formation, may delay the pathologic progression of AD via the Aβ clearance signaling pathway (Buxbaum and Johansson, 2017). A previous study showed that TTR concentration was substantially decreased in the peripheral blood of individuals with aMCI and AD, indicating that a set of sequester proteins, including TTR, can discriminate individuals with mild cognitive decline from healthy controls (Uchida et al., 2015). In contrast to this result, we found a significant increase in TTR concentration in AD patients when compared with normal controls, which might be attributed to the different ethnic populations revealing a number of influencing factors, such as heredity, environment, and lifestyle.

Among the candidate proteins, IGFBP2 was the one most frequently chosen. IGFBP-2 can influence DNA synthesis, cell proliferation and death, as well as glucose and amino acid uptake in cells by inhibiting IGF functions (Jones and Clemmons, 1995; Reyer et al., 2015). Overexpression of IGFBP-2 in mice led to decreased weights of the hippocampus, cerebellum, olfactory bulb, and prefrontal cortex (Schindler et al., 2017). In addition, IGFBP-2 was also reported to significantly increase in the serum of AD participants (Tham et al., 1993; McLimans et al., 2017), which was consistent with our findings. In addition, other insulin-like growth factors and the corresponding binding proteins were differentially expressed in both the CSF and serum from AD patients (Salehi et al., 2008), in which the expression of IGF-1 was reduced in serum (Vidal et al., 2016), while IGF-2 was highly expressed in CSF (Aberg et al., 2015). However, it has also been reported that serum, instead of CSF of IGF-1 and IGFBP3, was increased in AD (Johansson et al., 2013), both of which were more closely associated with AD in men than in women (Duron et al., 2012). Using a longitudinal case-control study, O’Bryant et al. (2010) found that serum protein-based biomarkers involving IGFBP-2 protein can be combined with clinical information to accurately classify AD (O’Bryant et al., 2010). Subsequently, using the multiplex panel, Doecke et al. (2012) identified an 18-plasma biomarker panel including IGFBP-2 that is useful for the diagnosis of AD (Doecke et al., 2012). These findings suggested that IGFBP-2 may be a key factor in a panel of protein biomarkers for the diagnosis of AD.

In addition, some proteins on the panel, including SAP, have been associated with hippocampal atrophy and the rate of change and progression to dementia (Thambisetty et al., 2010; Sattlecker et al., 2016). Importantly, we performed bioinformatics analysis and identified a close and interactive network among these proteins (data not shown), partly supporting the intimate relationship of the 8-protein panel with AD.

It is well known that recent clinical trials for the Aβ clearing strategies have failed (Galimberti and Scarpini, 2016; Mehta et al., 2017). Previous findings showed that 30% of cognitively normal elderly patients show signs of Aβ accumulation and, accordingly, a substantial number of AD patients show no signs of Aβ accumulation (Yang et al., 2012). In addition, several conflicting findings were demonstrated when using plasma Aβ peptides as markers for AD, suggesting that Aβ from peripheral blood may not reflect brain Aβ metabolism (Cummings, 2011; Hampel et al., 2012; Koyama et al., 2012). Importantly, Aβ can bind to a variety of proteins in blood, resulting in epitope masking and analytical interference (Marcello et al., 2009). In addition, methodology was also a limitation in the present study. The Milliplex MAP multiplex panels containing Aβ protein can only be used with CSF samples. To ensure the strong consistency of the results, the detection of plasma Aβ levels was not performed using other methods in this study.

Although many studies have identified plasma proteins related to AD [e.g., BDNF (Laske et al., 2007; Laske et al., 2011)], complement C4a (Bennett et al., 2012), IGFBP-2 (McLimans et al., 2017), TTR (Uchida et al., 2015), and SAP (Wilson et al., 2008), these are unlikely to be useful as a diagnostic test when used as single markers due to a lack of sensitivity and specificity. Based on the complexity of the pathogenesis of AD, as a relatively reliable biomarker for the diagnosis of AD, combinations of plasma proteins associated with various biological pathways may be necessary. Of note, accumulating evidence indicates that the replication and validation of results is urgent and important for exploratory studies. Thus, we plan to further validate our findings in a large-scale population that includes AD, MCI, and healthy controls.

The present findings should be interpreted considering some limitations. Since AD overlaps with other dementia forms, such as vascular dementia, frontotemporal dementia, and dementia with Lewy bodies, in the context of pathological traits, whether the same protein panel can accurately reflect their relationship to AD, but not to other dementia diseases, is a very important question. At present, only AD patients and healthy controls were included in this study, and no other dementia subtypes or disease stages were identified. In addition, our results may be influenced by the limited sample size. Moreover, due to the limitation of methodology, we did not detect plasma Aβ levels in the present study. Hence, more extensive studies including a large number of dementia subtypes should be conducted in the future. Furthermore, there are various factors, such as activity, diet, and medications, that can alter plasma protein levels.

## Conclusion

Taken together, the present study identified a plasma 8-protein panel including BDNF, AGT, IGFBP-2, OPN, cathepsin D, SAP, complement C4 and TTR that showed the highest determinative score for AD and healthy controls. Thus, these findings suggest that a combination of eight plasma proteins is a valuable diagnostic biomarker for AD in the Chinese population, providing novel insight for the diagnosis of AD.

## Author Contributions

ZC and SX conceived and designed the experiments. ZC, JY, HY, CJ, FZ, ZW, XL, YW, and TW performed the experiments and contributed to reagents, materials, and analysis tools. ZC, JY, HY, and CJ analyzed the data. ZC, JY, and SX wrote the paper.

## Conflict of Interest Statement

The authors declare that the research was conducted in the absence of any commercial or financial relationships that could be construed as a potential conflict of interest.
